# Effect of Caffeine and Other Methylxanthines on Aβ-Homeostasis in SH-SY5Y Cells

**DOI:** 10.3390/biom9110689

**Published:** 2019-11-02

**Authors:** Daniel Janitschke, Christopher Nelke, Anna Andrea Lauer, Liesa Regner, Jakob Winkler, Andrea Thiel, Heike Sabine Grimm, Tobias Hartmann, Marcus Otto Walter Grimm

**Affiliations:** 1 Experimental Neurology, Saarland University, 66424 Homburg/Saar, Germany; daniel.janitschke@uks.eu (D.J.); christophernelke@yahoo.de (C.N.); anna.lauer@uks.eu (A.A.L.); Liesa.regner@uks.eu (L.R.); Jakob.winkler@uks.eu (J.W.); andrea.thiel@uks.eu (A.T.); heike.grimm@gmx.de (H.S.G.); tobias.hartmann@uks.eu (T.H.); 2 Deutsches Institut für DemenzPrävention (DIDP), Saarland University, 66424 Homburg/Saar, Germany

**Keywords:** caffeine, theophylline, theobromine, propentofylline, pentoxifylline, methylxanthin, amyloid-β, amyloid precursor protein, Alzheimer’s disease, oxidative stress

## Abstract

Methylxanthines (MTX) are alkaloids derived from the purine-base xanthine. Whereas especially caffeine, the most prominent known MTX, has been formerly assessed to be detrimental, this point of view has changed substantially. MTXs are discussed to have beneficial properties in neurodegenerative diseases, however, the mechanisms of action are not completely understood. Here we investigate the effect of the naturally occurring caffeine, theobromine and theophylline and the synthetic propentofylline and pentoxifylline on processes involved in Alzheimer’s disease (AD). All MTXs decreased amyloid-β (Aβ) level by shifting the amyloid precursor protein (APP) processing from the Aβ-producing amyloidogenic to the non-amyloidogenic pathway. The α-secretase activity was elevated whereas β-secretase activity was decreased. Breaking down the molecular mechanism, caffeine increased protein stability of the major α-secretase ADAM10, downregulated *BACE1* expression and directly decreased β-secretase activity. Additionally, *APP* expression was reduced. In line with literature, MTXs reduced oxidative stress, decreased cholesterol and a decreased in Aβ1-42 aggregation. In conclusion, all MTXs act via the pleiotropic mechanism resulting in decreased Aβ and show beneficial properties with respect to AD in neuroblastoma cells. However, the observed effect strength was moderate, suggesting that MTXs should be integrated in a healthy diet rather than be used exclusively to treat or prevent AD.

## 1. Introduction

**Figure 1:**
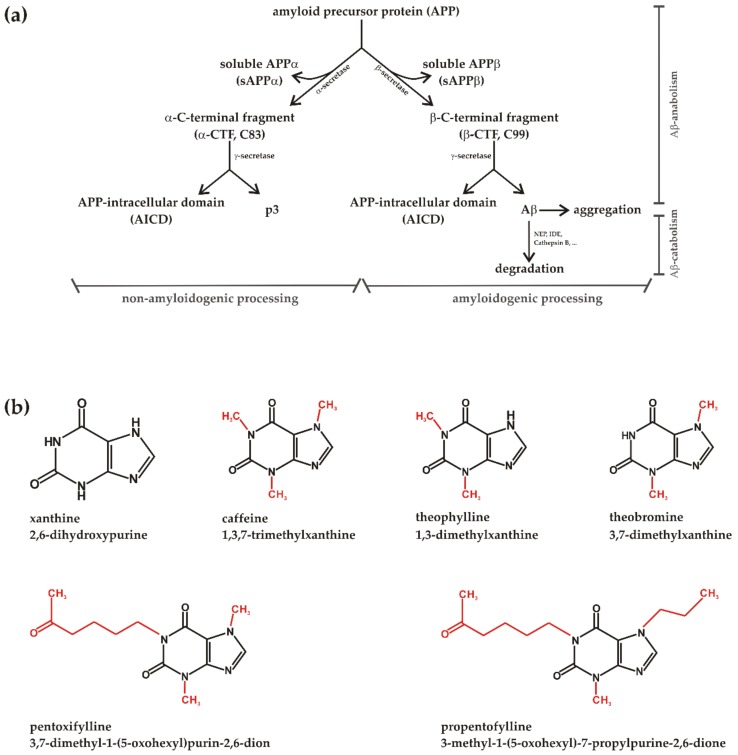
(**a**) Model of non-amyloidogenic and amyloidogenic processing of the amyloid precursor protein (APP) with subdivision into anabolism and catabolism of the neurotoxic APP cleavage product Aβ. The involved secretases and the resulting products are illustrated. (**b**) Chemical structures of xanthine (2,6-dihydroxypurine) and the analyzed methylxanthines (MTXs) caffeine (1,3,7-trimethylxanthine), theophylline (1,3-dimethylxanthine), theobromine (3,7-dimethylxanthine), pentoxifylline (3,7-dimethyl-1-(5-oxohexyl)purin-2,6-dion) and propentofylline (3-methyl-1-(5-oxohexyl)-7-propylpurine-2,6-dione). In red color the structural differences between the MTXs are highlighted.

Alzheimer’s disease (AD), the most common form of dementia in the elderly population, affects approximately 45 million people worldwide, emphasizing AD as a major public health concern [1,2]. Aging of the population is the main risk factor for suffering AD in industrialized nations (world health organization. www.who.int/ageing/publications/global_health) highlighting the need to delay the disease onset. AD is clinically characterized by degeneration of neurons in different brain regions, mainly cortical and subcortical areas as well as in the hippocampus, leading to deficits in memory, thinking and behavior [3]. Main histopathological hallmarks of AD are extracellular senile plaques composed of Aβ peptides and intracellular neurofibrillary tangles, consisting of hyperphosphorylated microtubule-associated tau proteins [4,5]. Aβ peptides are released by sequential proteolytic processing of the amyloid precursor protein (APP), a large type I transmembrane protein [6], by the action of the aspartylproteases β- and γ-secretase. The first step in Aβ generation is shedding of APP by β-secretase BACE1 [7,8,9], generating soluble secreted sAPPβ and a membrane-tethered C-terminal fragment, called β-CTF or C99 (Figure 1). The β-CTF is further processed by γ-secretase, generating Aβ peptides with variable C-terminus, mainly Aβ38, Aβ39, Aβ40 and Aβ42 peptides [10,11], and the APP intracellular domain (AICD) which is discussed to regulate gene transcription [12,13,14,15,16,17]. Aβ peptides can be either degraded mainly by neprilysin or insulin-degrading enzyme (IDE) [18,19], or can aggregate to amyloid senile plaques in the brains of individuals suffering from AD. The γ-secretase has been identified as a heterotetrameric protein complex, in which the presenilins (PS), either PS1 or PS2, constitute the catalytically active components [20,21]. Beside amyloidogenic APP processing involving β- and γ-secretase, APP can be cleaved by α-secretases, identified as members of the ADAM-family (a disintegrin and metalloproteinase), in a non-amyloidogenic pathway [22,23,24]. ADAM10 has been reported to be the main α-secretase in human brain [24]. The α-secretases process APP within the Aβ domain, thus precluding the formation of toxic Aβ peptides. Similar to β-secretase processing, α-secretase cleavage of APP in the ectodomain leads to a soluble α-secreted fragment (sAPPα), which is discussed to have neuroprotective functions [25,26,27], and to a C-terminal fragment called α-CTF or C83. This fragment is also further processed by the γ-secretase complex leading to non-toxic p3 peptides and AICD (Figure 1) [28]. Beside Aβ accumulation and tau hyperphosphorylation, multiple pathological processes are involved in AD pathogenesis, including e.g., inflammation, oxidative stress and changes in the energy- and lipid-metabolism [29,30,31]. However, despite intensive research, at present no drug is available to treat AD, illustrating the need to identify compounds that prevent AD or at least delay disease onset.

MTXs are alkaloids and a class of compounds derived from the purine xanthine. They have at least one methyl group at the nitrogen in the xanthine structure at R1, R2, or R3 (Figure 1). MTXs, especially caffeine, theobromine and theophylline are widely consumed all over the world being present e.g., in coffee, cola drinks, cacao or yerba mate. Whereas theobromine is abundant in chocolate, normally with a theobromine caffeine ratio greater than unity, caffeine is the main MTX in coffee and theophylline the major MTX in green tea [32,33]. MTXs are easily absorbed in the gastrointestinal tract and are able to cross the blood brain barrier. Besides their known psychostimulant actions MTXs have bronchiodilatory and bronchoprotective effects and are or have been used e.g., to treat asthma bronchiale, chronic bronchitis and emphysema. However, for bronchiodilatory effects high concentrations are needed, which are often associated with toxicity [34].

Interestingly, the critical view concerning the most popular MTX caffeine has changed dramatically. Whereas the intake of caffeine was assumed to be contraindicated or having adverse effects in many diseases, recent evidences show potential beneficial effects in neurodegenerative diseases like Parkinson’s disease or AD [35]. Several general molecular mechanisms of the action of MTXs are known, however, their exact beneficial impact in neurodegenerative diseases remain unclear. For example, it has been reported that MTXs, especially at higher dose, regulate intracellular calcium level [36,37], unselectively inhibits the ABCC4 and ABCC5 transporter [38], modulates GABA receptors or inhibit phosphodiesterase [39]. Moreover, at least some MTXs like caffeine or theophylline interact with DNA and modulate gene expression [40].

Due to their structural similarity to the purine adenosine, MTXs act as an antagonist of the adenosine receptor also known as purinergic P1 receptor, which is widely expressed in the body including the central nervous system [41]. Several neuroprotective aspects of MTXs can be explained by its adenosine receptor antagonism, which are discussed to result in a higher neuronal life span and neuronal activity: oxidative stress is decreased in presence of MTXs and inhibition of lipid peroxidation accompanied by an increase of antioxidant enzymes occurs [42], a property which is further enhanced by the direct scavenging properties of MTXs [43]. Furthermore, the inflammatory immune response can be modulated by inhibition of pro-inflammatory mediator release and attenuation of microglia cell activation [44].

Four adenosine receptor subtypes exist (A1, A2A, A2B, A3) in humans. Interestingly, theobromine lacking the methyl group at the R1 position has only a weak activity as adenosine receptor antagonist whereas an elongation of the methyl group in R1 as seen in pentoxifylline and propentofylline generally increases the affinity to the adenosine receptor [45]. In addition, the MTXs vary in their affinity to the receptor subtype and some properties of MTXs seem to be independent of the adenosine receptor and specific for single MTXs. For example, caffeine but no other MTXs was able to inhibit inositol 1,4,5-trisphosphate receptor mediated calcium release in acinar cells in an experimental acute pancreatitis model suggesting an adenosine receptor independent function of caffeine [46].

Therefore, the aim of the study was to (I) further elucidate the molecular mechanism especially of caffeine on the protective effect in respect to AD focusing on the Aβ metabolism and (II) compare these effects with the other two mainly naturally occurring MTXs theophylline and theobromine and two synthetic MTXs propentofylline and pentoxifylline widely used as a drug having a higher affinity to adenosine receptors compared to the naturally occurring MTXs.

## 2. Materials and Methods 

### 2.1. Chemicals and Reagents

Caffeine and theophylline were purchased from Fisher Scientific (Schwerte, Germany), theobromine, pentoxifylline, propentofylline and all other chemicals used in this study were acquired from Merck former Sigma-Aldrich (Darmstadt, Germany), if not stated otherwise.

### 2.2. Cell Culture

For all cell-based experiments human neuroblastoma SH-SY5Y wildtype (wt) cells were cultivated in Dulbecco’s Modified Eagle’s Medium (DMEM), containing 10% fetal calf serum (FCS, GE Healthcare Life Sciences, Chalfont St. Giles, United Kingdom) and 0.1% non-essential amino acid solution (MEM). For the stable transfected cell line SH-SY5Y APP^695^, which overexpressed the human mainly neuronal APP isoform APP^695^ (see Supplement Figure S1), 0.3 mg/mL hygromycin B (PAN-Biotech, Aidenbach, Germany) was added to the medium. For the murine neuroblastoma cell line N2a, culture medium contained DMEM, 10% FCS, 0.1% MEM and was supplemented with penicillin/streptomycin solution, 1 mM sodium-pyruvate, and 2 mM l-glutamine.

### 2.3. Methylxanthines Incubations

#### 2.3.1. Cell Culture

FCS in DMEM was reduced to 0.1%, dependent on the following experiments, 16 h prior incubation. FCS was reduced to elucidate the potential effect of MTXs on cholesterol or other lipids being also present in FCS. MTXs were incubated with a concentration of 100 µM for 24 h (8 + 16 h), whereas controls were treated with HPLC-grade H_2_O (Fisher Scientific, Schwerte, Germany) as a solvent control. For experiments with γ-secretase inhibitor IX, cells were pretreated 2 h with 2.5 µM γ-secretase inhibitor, before incubation with MTXs was performed as described above. For determination of cholesterol level, cells were long-term incubated for six days in DMEM reduced to 1% FCS.

#### 2.3.2. Cell Lysates

Cells were lysed by using 150 mM NaCl (VWR Chemicals, Radnor, PA, USA), 50 mM Tris/HCl pH 7.4, 2 mM EDTA (Carl Roth, Karlsruhe, Germany), 1% NP-40, 1% Triton-X 100 lysis buffer supplemented with protease inhibitor (Roche Diagnostics, Risch-Rotkreuz, Switzerland). Subsequently, the protein concentrations of the lysates were determined by bicinchoninic acid assay (BCA) as described [47] and adjusted to equal protein concentrations for following experiments.

#### 2.3.3. Cell Homogenates

Cultivated SH-SY5Y wt cells were washed two times with ice-cold PBS and collected in 150 µL HPLC-grade H_2_O. The solution was then homogenized via Minilys (Peqlab, Erlangen, Germany) for 90 s on maximum intensity. Homogenates were adjusted to 10 mg/mL by BCA (see 2.3.2.) and stored at −80 °C.

#### 2.3.4. Postnuclear Fractions 

Postnuclear fractions (PNFs) of SH-SY5Y wt cells were obtained by washing cells three times with ice-cold PBS and homogenizing in sucrose-buffer (200 mM sucrose, 10 mM Tris/HCl pH 7.4, β-sucrose buffer additionally 1 mM EDTA) via Minilys (Peqlab, Erlangen, Germany) on maximum intensity for 20 s. For the preparation of mouse brain PNFs the brains from male C57BL/6 wt mice, which were kindly provided by Prof. Dr. med. Matthias W. Laschke and frozen in liquid nitrogen directly after removing, were slowly defrosted on ice and homogenized in the same way described for SH-SY5Y wt cells before. Afterwards protein amount was adjusted to same concentration by BCA (see 2.3.2.). Samples were then centrifuged at 900 rcf for 10 min at 4 °C and supernatants were collected and stored at −80 °C. PNFs were incubated with MTXs at a concentration of 0.1 nmol MTX per 1 µg protein for 30 min before α- and β-secretase activity measurements.

### 2.4. Western Blot (WB) Experiments

After BCA protein adjustment, the level of APP, ADAM10, and β-CTF were analyzed in cell lysates whereas for the determination of the total protein level of the secreted proteins Aβ, sAPPα, and sAPPβ growth media were used. The following additional loading controls were used to determine whether potential differences were due to alterations in protein loading of the western blot (WB): For secreted proteins medium was supplemented with bovine serum albumin (BSA) and detected via Ponceau staining as described in [48]. In lysates actin WB was used as a loading control (anti-actin antibody ab1801 (1:1000; abcam, Cambridge, UK) [49]. For immune precipitation experiments Immunoglobulin G (IgG) antibody signals were quantified and used as an internal loading control as described in [50]. In all cases no significant correlation was found between loading control, and we observed no effect of the protein of interest. No significant differences in the loading control of treated and untreated samples or cells were observed. Antibodies and dilutions that were used in this study are: W02 antibody for the detection of APP, β-CTF, total Aβ and sAPPα (5 µg/mL; Millipore, Billerica, MA, USA), anti-ADAM10 735-749 Rabbit pAb (1:2000; Merck, Darmstadt, Germany), anti-Human sAPPβ 18957 (1:50; IBL America, Minneapolis, MN, USA), anti-rabbit IgG HRP Conjugate W401B (1:5000; Promega, Mannheim, Germany) and anti-mouse P0260 (Dako, Hamburg, Germany). To detect proteins the enhanced chemiluminescense (ECL)-method (Perkin Elmer, Rodgau-Jügesheim, Germany) was used. Densitometrically quantification was performed with Image Gauge V3.45 software (Fujifilm, Düsseldorf, Germany).

### 2.5. Immunoprecipitation

Aβ, and β-CTF level were detected by performing immunoprecipitation of equal volumes of conditioned media or cell lysates adjusted to the same protein amount as described in Grimm et al. [51]. Precipitates were then used for WB experiment as described above (see 2.4.).

### 2.6. Determination of Total Aβ-Degradation in N2a Wt Cells

To determine the total degradation of Aβ, human synthetic Aβ40 peptide (Bachem, Bubendorf, Switzerland) was supplemented (0.5 µg/mL) for an additional 6 h after cultivation of murine N2a wt cells in reduced FCS (0.1%)/DMEM for 6 h and subsequent incubation of 100 µM MTXs or HPLC-grade H_2_O as control for 18 h. Afterwards, non-degraded human Aβ was detected by WB analysis using W02 antibody.

### 2.7. Secretase Activity Assays

#### 2.7.1. α-, β-, γ-Secretase Activity in Living SH-SY5Y Wt Cells 

The activities of α-, β- and γ-secretases in living cells were analyzed as described in Grimm et al. [52].

#### 2.7.2. α-, β- Secretase Activity on Postnuclear, Cell Free Fractions

Activities of α- and β- secretase in postnuclear, cell free fractions were analyzed as described in Grimm et al. [53].

### 2.8. Quantitative Real-Time Polymerase Chain Reaction (RT-PCR) Experiments

Gene expression analysis was performed as described in Grimm et al. [54] and the following primers were used. TATA-box binding protein (*TBP*): forward 5′-CGG AGA GTT CTG GGA TTG T-3′, reverse 5′-GGT TCG TGG CTC TCT TAT C-3′; amyloid precursor protein (*APP*): forward 5′-GGC AGT TAT CCA GCA TTT CC-3′, reverse 5′-ATT GAG CAT GGC TTC CAC TC-3′; β-site APP-cleaving enzyme 1 (*BACE1*): forward 5´-GCA GGG CTA CTA CGT GGA GA-3´, reverse 5′-TAG TAG CGA TGC AGG AAG GG-3′; a disintegrin and metalloproteinase domain-containing protein 10 (*ADAM10*): forward 5′-GCA AAC TGA AAC CTG GGA AA-3′, reverse 5′-TTC CTT CCC TTG CAC AGT CT-3′ (Eurofins MWG Operon, Eberberg, Germany). Results were normalized to TBP and the 2^-(ΔΔCt)^ method was used to calculate expression changes.

### 2.9. Protein Stability of ADAM10

SH-SY5Y wt cells were cultivated in DMEM containing FCS (0.1%) for 16 h and then incubated for 8 + 16 h with 100 µM caffeine or HPLC-grade H_2_O. After incubation, cells were treated with 100 µM of cycloheximide for 1 h before it was replaced by incubation media also containing cycloheximide. Cells with treatment of cycloheximide were then collected after 6 h and 12 h. ADAM10 protein was detected via WB experiment as described above (see 2.4.).

### 2.10. Detection of Reactive Oxygen Species (Hydroxyl Radical, Hypochlorite, Peroxynitrite)

Incubated cells on a 96 well plate were washed twice with prewarmed (37 °C) imaging solution (0.9% NaCl, 20 mM HEPES, pH 7.4). Afterwards, 100 µL imaging solution containing 10 µM 3′-(p-aminophenyl)fluorescein (APF; Invitrogen, Carlsbad, CA, USA) were added per well. Fluorescence was measured directly after APF treatment, and after 1 h incubation in the dark at 37 °C, at excitation wavelength 490 nm ± 10 nm and emission wavelength of 515 ± 10 nm using a Safire^2^ Fluorometer (Tecan, Crailsheim, Germany).

### 2.11. Cholesterol Concentration

The amount of cholesterol was measured from cell homogenates by using the Amplex Red Cholesterol Assay Kit (Invitrogen, Carlsbad, CA, USA) according to the manufacturer’s protocol.

### 2.12. Aβ Aggregation Via Thioflavin T Assay

Aβ aggregation via Thioflavin T assay was done as described before [51] with small modifications. After monomerization 1µM Aβ42 was used to determine potential differences between control and 100 µM MTXs. As an additional control epigallocatechin gallate (EGCG) was used, as EGCG is known to be a potent inhibitor of Aβ aggregation [55]. All experiments (MTXs, control and EGCG) were performed in the presence of 10 µM phosphatidylcholine 16:0.

### 2.13. Lactate Dehydrogenase (LDH) Activity Assay

To measure the cytotoxicity of the MTXs incubation, Cytotoxicity Detection Kit (LDH) from Roche (Basel, Switzerland) was used according to the manufacturer’s instructions.

### 2.14. Cell Proliferation (XTT) Assay

For the determination of differences in cell proliferation dependent on particular MTX incubation, the cell proliferation II Kit (XTT) from Roche (Basel, Switzerland) was used according to the protocol of the manufacturer.

### 2.15. Statistical Analysis

All quantified data represent an average of at least three independent experiments. To compare the effect of caffeine with the other MTXs the average effect of all MTXs was calculated. Therefore the sum of the individual effects of the MTXs compared to solvent control was calculated and divided by the number of different MTXs (five: caffeine, theophylline, theobromine, pentoxifylline, propentofylline) revealing the average effect strength of all MTXs. The individual effects of all MTXs are shown in the supplemental data. Error bars represent standard deviation of the mean. Statistical significance was determined by two-tailed Student’s t test for comparing two parameters. For multiple parameter comparison ANOVA, and Tukey-HSD post hoc test were used. Significance was set at * *p* ≤ 0.05, ** *p* ≤ 0.01, and *** *p* ≤ 0.001.

## 3. Results

### 3.1. Influence of Methylxanthines on Total Aβ Level

**Figure 2:**
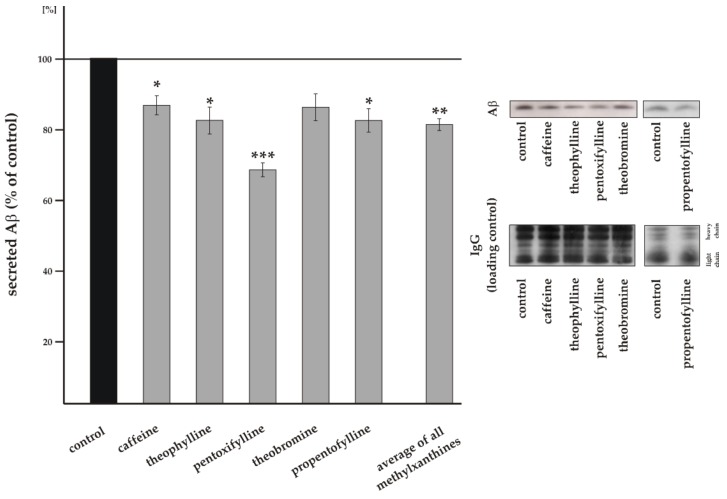
Effect of MTXs on Aβ generation. Level of total secreted Aβ in the medium of treated SH-SY5Y amyloid precursor protein (APP)^695^ cells. Treatment with ddH_2_O as solvent control was set to 100%. Representative Aβ signals after immunodetection and the corresponding IgG signals as loading control are shown on the right side of the figure. Error bars represent the standard error of the mean. Asterisks show the statistical significance calculated by unpaired Student’s t test (* *p* ≤ 0.05; ** *p* ≤ 0.01; *** *p* ≤ 0.001). No significant differences were found between the effect strengths of the analyzed MTXs using ANOVA analysis.

To analyze whether MTXs influence total Aβ level, we used the neuroblastoma cell line SH-SY5Y stably transfected with APP^695^, the main APP isoform in neurons [56]. Cells were incubated for 8 + 16 h with either ddH_2_O as solvent control or caffeine, theophylline, pentoxifylline, theobromine or propentofylline in a final concentration of 100 µM. Under these conditions both cell proliferation, determined by XTT assay, and toxicity, measured by LDH release, were not affected. Effects on cell proliferation compared to control were <4% and for all MTXs not statistically significant. Cell viability differences compared to control were <4% also lacking statistically significance for all MTXs (see Supplemental Table S1). Total secreted Aβ level were determined by the use of the antibody W02, detecting Aβ peptides with variable C-terminus (Aβ1-x), including Aβ40 and Aβ42 peptides. As shown in Figure 2 all investigated MTXs reduced the total secreted Aβ level in a similar range. In line with current literature describing impaired Aβ generation in presence of caffeine [57,58], caffeine significantly reduced secreted Aβ level to 84.5% ± 5.4% (*p* = 0.0205) in our cellular system (Figure 2). A comparable protective effect regarding total Aβ level was obtained for theophylline, theobromine and propentofylline. Theophylline significantly reduced secreted Aβ level to 80.2% ± 7.6% (*p* = 0.0310), theobromine to 83.9% ± 7.7% (*p* = 0.0730), although the observed decrease was not statistically significant and propentofylline incubated cells showed identical to theophylline a significant reduction to 80.2% ± 6.6% (*p* = 0.0168). The strongest reduction in total secreted Aβ level was obtained for pentoxifylline: 66.2% ± 3.9% (*p* ≤ 0.001). Averaged, all analyzed MTXs significantly reduced total Aβ level to 78.9% ± 3.3% (*p* = 0.0032) (Figure 2), indicating potential beneficial effects of MTXs concerning total Aβ level. A reduction in total Aβ level can be caused either by an altered Aβ anabolism affecting amyloidogenic and/or non-amyloidogenic APP processing or by an altered Aβ catabolism. In order to investigate whether the reduced Aβ level in presence of MTXs are caused by a changed non-amyloidogenic APP cleavage preventing the formation of Aβ peptides, we next analyzed α-secretase cleavage in presence of MTXs.

### 3.2. Methylxanthines Increase Non-Amyloidogenic α-Secretase Cleavage of APP 

**Figure 3:**
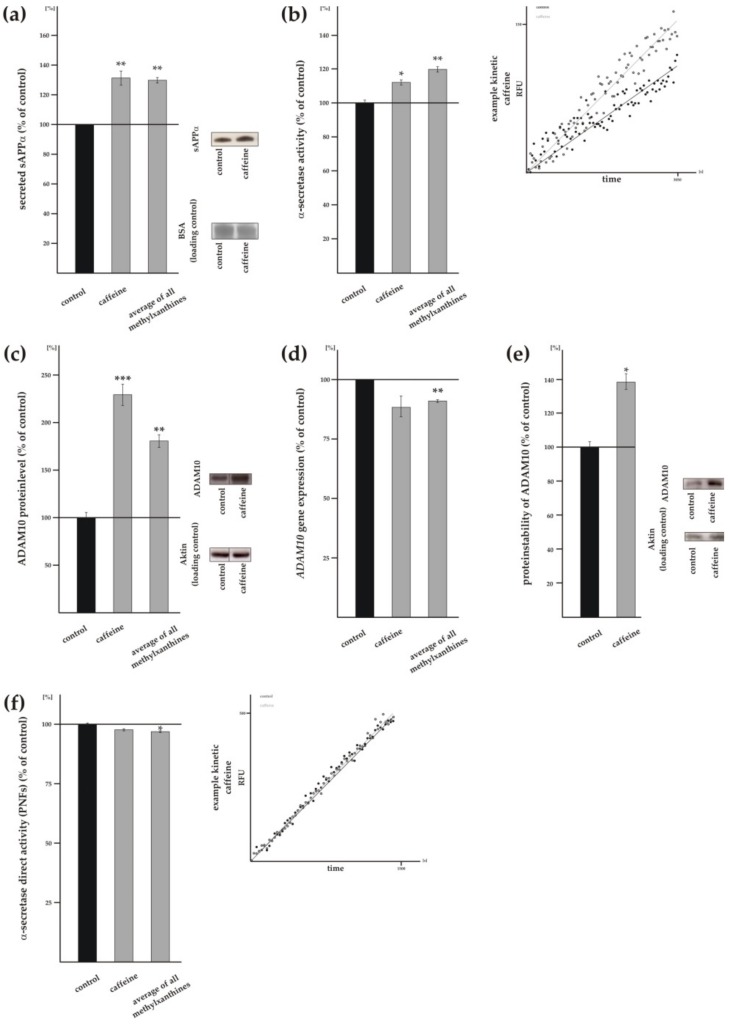
Effects of caffeine and other MTXs on non-amyloidogenic APP processing. (**a**) Secreted soluble sAPPα of treated SH-SY5Y cells (*n* ≥ 9) and representative western blots (WBs) including load control. (**b**) Analysis of the α-secretase activity in living SH-SH5Y cells after treatment with MTXs (*n* ≥ 4). A representative kinetic of caffeine is shown in the right part of the figure. (**c**) Protein level of ADAM10 (*n* ≥ 4) and representative WBs including load control. (**d**) RT-PCR analysis of *ADAM10* compared to control conditions (*n* ≥ 10). (**e**) Determination of ADAM10 degradation (*n* ≥ 4). Representative WBs including load control are shown on the right. (**f**) α-secretase activity in post nuclear fractions of incubated SH-SY5Y cells (*n* ≥ 4) and representative kinetic after treatment with caffeine. Error bars represent the standard error of the mean. Asterisks show the statistical significance calculated by unpaired Student’s t test (* *p* ≤ 0.05; ** *p* ≤ 0.01; *** *p* ≤ 0.001).

The determination of α-secreted APP (sAPPα) in presence of MTXs was performed in SH-SY5Y cells stably expressing APP^695^. WB analysis of sAPPα using the antibody W02 showed that caffeine significantly elevated sAPPα level to 131.3% ± 9.5% (*p* = 0.0070). Nearly identical to caffeine, all analyzed MTXs averaged revealed a significant increase in sAPPα level to 129.8% ± 3.7% (*p* = 0.0013) (Figure 3a). In detail, theophylline and propentofylline significantly elevated sAPPα level to 122.8% ± 8.8% (*p* = 0.0256) and 143.1% ± 13.3% (*p* = 0.0117), respectively (Supplement Figure S2a). Pentoxifylline and theobromine also increased sAPPα level, however statistical analysis revealed no significance (pentoxifylline: 129.2% ± 14.8%, *p* = 0.0741; theobromine: 122.8% ± 16.3%, *p* = 0.1915). To further elucidate non-amyloidogenic APP processing in presence of MTXs we measured α-secretase activity in living SH-SY5Y wt cells using a fluorescence-based assay. In line with the observed increase in sAPPα level, caffeine as well as all other tested MTXs significantly increased α-secretase activity. Caffeine significantly elevated α-secretase activity to 112.3% ± 3.0% (*p* = 0.0195), all MTXs averaged showed a significant increase to 120.0% ± 3.2% (*p* = 0.0035) (Figure 3b). Thus, theophylline significantly increased α-secretase activity to 122.1% ± 3.8% (*p* = 0.0026), pentoxifylline to 114.9% ± 5.2% (*p* = 0.0385), theobromine to 130.9% ± 1.1% (*p* ≤ 0.001) and propentofylline to 119.6% ± 4.9% (*p* = 0.0021) (Supplement Figure S2b). The increase in sAPPα level as well as in α-secretase activity in presence of MTXs indicate that MTXs decrease total secreted Aβ level by an elevation of non-amyloidogenic α-secretase APP processing. As we measured α-secretase activity in living cells, the observed increase in α-secretase shedding of APP can be caused by direct and/or indirect effects of MTXs, like e.g., gene/protein expression, protein transport or protein stability. Therefore we next analyzed ADAM10 protein level, the physiologically relevant constitutive α-secretase in neurons [24]. Caffeine-incubated SH-SY5Y wt cells showed a significant increase in ADAM10 protein level to 229.3% ± 22.4% (*p* ≤ 0.001) (Figure 3c) and the average of all MTXs also revealed a significant increase to 180.7% ± 13.3% (*p* = 0.0037). In detail, all analyzed MTXs individually significantly elevated the protein level of ADAM10 (theophylline: 177.0 ± 18.1%, *p* = 0.0037; pentoxifylline: 179.5% ± 17.9%, *p* = 0.0028; theobromine: 148.8% ± 8.1%, *p* = 0.0024; propentofylline: 168.8% ± 9.8%, *p* = 0.204) (Supplement Figure S2c). To examine whether the MTX-induced elevation of the ADAM10 protein level is caused by an increase in *ADAM10* gene expression, we performed RT-PCR analysis of *ADAM10* in MTX-incubated SH-SY5Y wt cells. Caffeine slightly but not significantly reduced *ADAM10* gene expression (caffeine: 88.4% ± 8.8%, *p* = 0.2186) (Figure 3d). Theophylline, pentoxifylline, theobromine and propentofylline also slightly but not significantly decreased *ADAM10* gene expression (theophylline: 89.4% ± 4.6%, *p* = 0.0876; pentoxifylline: 92.9% ± 4.5%, *p* = 0.1067; theobromine: 89.7% ± 6.1%, *p* = 0.1226; propentofylline: 94.5% ± 8.5%, *p* = 0.5321) (Supplement Figure S2d). Caused by the consistent slight decrease in *ADAM10* gene expression of the analyzed MTXs, all MTXs averaged revealed a slight but significant reduction in *ADAM10* RNA level to 90.9% ± 1.2% (*p* = 0.0015) (Figure 3d). These results indicate that the observed increase in ADAM10 protein level in presence of MTXs is not caused by an elevation in *ADAM10* gene expression, but rather by an influence of MTXs on ADAM10 protein stability. To analyze the effect of MTXs on ADAM10 protein stability we selected caffeine, as caffeine showed the strongest effect on ADAM10 protein level. Protein stability was determined by cycloheximide treatment in SH-SY5Y wt cells. Compared to control cells, in presence of caffeine ADAM10 protein level was significantly elevated to 138.5% ± 9.2% (*p* = 0.0347), indicating that caffeine elevates ADAM10 protein stability (Figure 3e).

Beside the indirect effect of MTXs on non-amyloidogenic APP processing, MTXs might also directly affect α-secretase activity. To evaluate whether MTXs have a direct effect on α-secretase activity we prepared post nuclear fractions of SH-SY5Y wt cells, incubated them with MTXs in a cell free assay and measured α-secretase activity. Caffeine (Figure 3f) as well as theophylline, pentoxifylline, theobromine and propentofylline (Supplement Figure S2e) slightly but not significantly reduced α-secretase activity (caffeine: 97.7% ± 0.9%, *p* = 0.1790; theophylline: 97.5% ± 3.2%, *p* = 0.4904; pentoxifylline: 93.6% ± 2.4%, *p* = 0.0628; theobromine: 97.9% ± 3.1%, *p* = 0.5708; propentofylline: 97.6% ± 2.3%, *p* = 0.3980). However, as the analyzed MTXs consistently showed a slight reduction, the average of all MTXs revealed a minor but significant direct effect on α-secretase activity (MTXs averaged: 96.9% ± 0.8%, *p* = 0.0159) (Figure 3f). However, as the observed effect is not significant for the individual MTXs and the average effect strength is 3.1%, a direct effect on α-secretase activity does not mainly influence the increased α-secretase processing in presence of MTXs.

### 3.3. Influence of Methylxanthines on Amyloidogenic APP Processing

**Figure 4:**
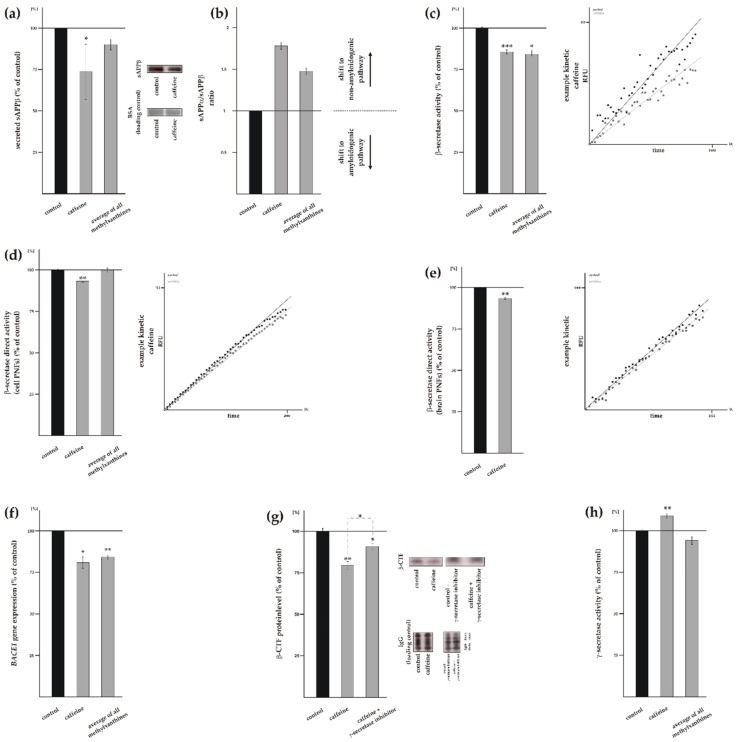
Effect of caffeine and other MTXs on amyloidogenic processing of APP. (**a**) Protein level of sAPPβ in caffeine treated cells compared to solvent control (*n* ≥ 13). Representative WBs including load control are shown on the right. (**b**) sAPPα/sAPPβ ratio. (**c**) Activity of the β-secretase on living cells (*n* ≥ 19). A representative kinetic of caffeine is shown on the right. (**d**) β-secretase activity in cell free post nuclear fractions of treated SH-SY5Y wt cells (*n* ≥ 6). (**e**) Activity of β-secretase in caffeine-treated post nuclear fractions of wt mouse brain (*n =* 6). Kinetics of β-secretase activity in incubated post nuclear fractions are illustrated on the right side. (**f**) Expression of *BACE1* in cells treated with MTXs (*n* ≥ 10). (**g**) Protein level of β-C-terminal fragment (*n* ≥ 4). A significant difference in the effect strength between caffeine and the average of all MTXs was found. (**h**) Analysis of the γ-secretase on living cells after caffeine treatment (*n* ≥ 25). Error bars represent the standard error of the mean. Asterisks show the statistical significance calculated by unpaired Student’s t test (* *p* ≤ 0.05; ** *p* ≤ 0.01; *** *p* ≤ 0.001).

Beside the positive effect of MTXs on non-amyloidogenic APP processing preventing the formation of Aβ, a reduction of total Aβ level can be also caused by an impaired amyloidogenic APP processing. To investigate the effect of MTXs on amyloidogenic β-secretase mediated APP processing, we first determined the sAPPβ level by the use of a specific sAPPβ antibody. WB analysis of sAPPβ revealed a significant reduction in the sAPPβ level in presence of caffeine to 73.7% ± 8.9% (*p* = 0.0110) (Figure 4a). The sAPPβ level was also significantly reduced in presence of pentoxifylline and propentofylline (pentoxifylline: 82.3% ± 7.2%, *p* = 0.0298; propentofylline: 86.0% ± 3.1%, *p* ≤ 0.001) (Supplement Figure S3a), whereas theophylline and theobromine showed no significant effect (theophylline: 109.7% ± 9.2%, *p* = 0.3085; theobromine: 98.7% ± 8.2%, *p* = 0.8765). Averaged, all tested MTXs tended to decrease the sAPPβ level, however, statistical analysis did not reach significance, as two out of five MTXs showed no effect on the sAPPβ level. This might be due to the fact that the observed effect strength is rater small and WB analysis of sAPPβ might not be sensitive enough to get statistical significant results. Furthermore the ratio of sAPPα/sAPPβ was calculated, because it represents the shift from amyloidogenic to non-amyloidogenic pathway (Figure 4b, Supplement Figure S3b). To further determine whether a reduced amyloidogenic APP processing contributes to the reduced total Aβ level in presence of MTXs, we examined β-secretase activity in living SH-SY5Y wt cells. Consistently, caffeine (Figure 4c) as well as theophylline, pentoxifylline, and propentofylline (Supplement Figure S3c) significantly reduced β-secretase activity (caffeine: 85.7% ± 2.3%, *p* ≤ 0.001; theophylline: 86.4% ± 3.0%, *p* ≤ 0.001; pentoxifylline: 79.9% ± 2.8%, *p* ≤ 0.001; propentofylline: 74.0% ± 2.7%, *p* ≤ 0.001), whereas theobromine showed no effect on β-secretase activity in living cells: 95.2% ± 2.6% (*p* = 0.1051) (Supplement Figure S3c). Averaged, all MTXs showed a significant reduction in β-secretase activity to 84.2% ± 2.3% (*p* = 0.011) (Figure 4c), indicating that MTXs also reduce Aβ generation by decreasing amyloidogenic β-secretase processing. In contrast to the results showing no direct effect of caffeine on α-secretase activity, we obtained a significant reduction to 94.2% ± 1.1% (*p* = 0.0083), when post nuclear fraction of human neuroblastoma cells were incubated with caffeine and β-secretase activity was determined in a cell free assay (Figure 4d). However, all MTXs averaged showed no direct effect on β-secretase activity (Figure 4d) as theophylline, pentoxifylline and theobromine showed no effect, whereas propentofylline even increased β-secretase activity directly (Supplement Figure S3d) revealing that MTXs have no homogenous effect in respect to a direct effect on β-secretase activity. In line with cell culture experiments, post-nuclear fractions of wildtype mouse brain treated with caffeine revealed a direct effect and resulted in a slight but significantly reduced activity of β-secretase to 93.6% ± 1.0% (*p* = 0.0032) (Figure 4e). To further elucidate whether MTXs affect β-secretase cleavage indirectly, we determined mRNA level of *BACE1* in SH-SY5Y wt cells by RT-PCR. *BACE1* gene expression was significantly reduced to 80.8% ± 6.8% (*p* = 0.0204) in presence of caffeine and the average of all MTXs also showed a significant reduction in *BACE1* mRNA level to 84.2% ± 2.7% (*p* = 0.0043) (Figure 4f). In detail, theophylline, pentoxifylline and theobromine highly significantly reduced *BACE1* gene expression in a similar range to approximately 80% (theophylline: 82.1% ± 4.9%, *p* ≤ 0.001; pentoxifylline: 82.2% ± 4.4%, *p* ≤ 0.001; theobromine: 77.8% ± 3.7%, *p* ≤ 0.001) whereas propentofylline only trended to decrease *BACE1* gene transcription (Supplement Figure S3e). To validate the observed effect of MTXs on amyloidogenic APP processing, we determined the level of β-CTF for caffeine in presence and absence of a γ-secretase inhibitor. The β-CTF level was significantly reduced to 79.5% ± 2.7% (*p* = 0.0040) in caffeine-incubated SH-SY5Y cell stably expressing APP^695^ in the absence of γ-secretase inhibitor (Figure 4g). When further processing of β-CTF was blocked by a γ-secretase inhibitor in caffeine-incubated cells, we observed a significant reduction to 90.9% ± 2.0% (*p* = 0.0307) compared to MTX untreated cells (Figure 4g). The different effect strength of caffeine on β-CTF in presence or absence of γ-secretase inhibitor suggests that γ-secretase activity might also be affected by MTX. We therefore analyzed γ-secretase activity, which is crucial for the release of Aβ. The determination of γ-secretase activity in living cells revealed a slight but significant increase in γ-secretase activity to 108.9% ± 2.7% (*p* = 0.0062) in presence of caffeine whereas the average of all MTXs trended to decrease γ-secretase activity (MTXs averaged: 94.1% ± 4.5%, *p* = 0.2629) (Figure 4h). Individually, theobromine and propentofylline significantly reduced γ-secretase activity to 84.6% ± 2.9% (*p* ≤ 0.001) and 84.7% ± 4.6% (*p* ≤ 0.001), respectively, whereas theophylline and pentoxifylline showed no significant reduction (Supplemental Figure S3e).

In summary, MTXs in general slightly decrease the amyloidogenic pathway by decreasing β- and/or γ-secretase activity. Interestingly, the effect on the amyloidogenic pathway is not so pronounced compared to the non-amyloidogenic pathway. Moreover, the underlying effects and mechanisms in the amyloidogenic pathway are not conserved for all MTXs. However the combined effects of both amyloidogenic and non-amyloidogenic pathways result finally for all MTXs in a consistent Aβ decrease, where no statistical significant differences between the single MTXs were observed.

### 3.4. Influence of Methylxanthines on Additional Cellular Processes Involved in Aβ Homeostasis

**Figure 5:**
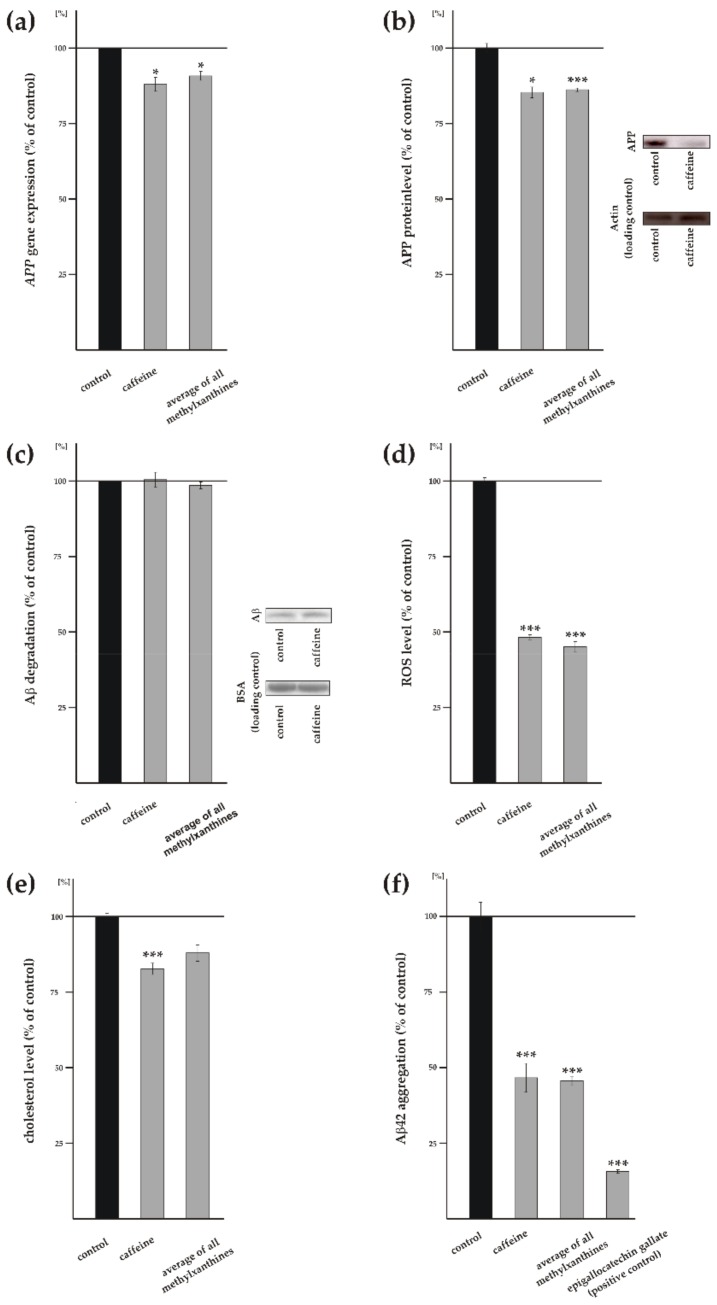
Effects of MTXs on APP level, Aβ catabolism and aggregation, reactive oxygen species and cholesterol. (**a**) Gene expression of *APP* was analyzed by RT-PCR (*n* ≥ 10). (**b**) Protein level of APP (*n* ≥ 9). Representative WBs including load control are shown on the right. (**c**) Degradation of Aβ (*n* ≥ 11). Representative WBs including load control are shown on the right. (**d**) Influence of MTXs on reactive oxygen species (ROS) level (hydroxyl radical, hypochlorite, peroxynitrite) (*n* ≥ 8). (**e**) Level of cholesterol in treated human neuroblastoma cells (*n* ≥ 4). (**f**) Effect of caffeine and MTXs on aggregation of Aβ42 (*n* ≥ 20). Epigallocatechin gallate (EGCG) served as positive control. Error bars represent the standard error of the mean. Asterisks show the statistical significance calculated by unpaired Student’s t test (* *p* ≤ 0.05; ** *p* ≤ 0.01; *** *p* ≤ 0.001).

Total Aβ level and AD pathogenesis are not only dependent on amyloidogenic and non-amyloidogenic APP processing, but rather are determined by additional cellular processes involved in Aβ homeostasis like e.g., gene and protein expression of the Aβ precursor APP, degradation of Aβ peptides and the level of reactive oxygen species (ROS) and of cholesterol. Gene expression of *APP* was significantly reduced to 88% ± 4.6% (*p* = 0.0280) in caffeine-treated SH-SY5Y wt cells (Figure 5a). The average of all analyzed MTXs also revealed a significant reduction in *APP* gene transcription to 90.8% ± 2.9% (*p* = 0.0349). Besides propentofylline, all tested MTXs individually significantly reduced *APP* gene transcription (theophylline: 83.2% ± 3.2%, *p* ≤ 0.001; pentoxifylline: 93.3% ± 2.7%, *p* = 0.0148; theobromine: 88.8% ± 2.5%, *p* = 0.0015; propentofylline: 100.6% ± 13.0%, *p* = 0.9614) (Supplement Figure S4a). In line with these results, protein level of APP was also significantly reduced to 85.4% ± 3.6% (*p* = 0.0118) in presence of caffeine (Figure 5b). Similarly, the average of all MTXs revealed a significant reduction of the *APP* protein level to 86.2% ± 1.2% (*p* ≤ 0.001), which is also in line with the observed reduced APP gene transcription. In detail, theophylline and pentoxifylline showed a reduction to 90.5% ± 4.2% (*p* = 0.1027) and 83.7% ± 6.7% (*p* = 0.0511), respectively (Supplement Figure S4b). However, statistical analysis did not reach significance. A significant reduction of the APP protein level was obtained for theobromine and propentofylline that reduced APP protein level to approximately 86% (theobromine: 85.5% ± 5.7%, *p* = 0.0483; propentofylline: 86.1% ± 5.0%, *p* = 0.0437) (Supplement Figure S4b). These results indicate that MTXs also reduce total Aβ level by a decrease in *APP* gene expression accompanied by reduced APP protein level.

Beside the above addressed Aβ anabolism, including APP processing, the amount of Aβ strongly depends on Aβ catabolism via the main Aβ-degrading enzymes neprilysin and IDE [18,19]. In order to investigate whether MTXs affect the degradation of Aβ peptides, we used mouse neuroblastoma N2a cells and treated these cells with MTXs in presence of human synthetic Aβ peptides. Remaining synthetic Aβ peptides were detected by WB analysis using the antibody W02 recognizing human but not endogenous mouse Aβ. Caffeine as well as all MTXs individually and averaged showed no effect on remaining Aβ peptides and thus Aβ degradation (Figure 5c and Supplement Figure S4c), indicating that Aβ degradation is not affected by MTXs.

Oxidative stress is also known to play an important role in the pathogenesis of AD. To analyze whether MTXs affect the production of ROS, we used SH-SY5Y wt cells and APF to selectively detect highly reactive oxygen species (hROS), including hypochlorite and free hydroxyl radicals [59]. The production of ROS was significantly reduced to 48.3% ± 1.6% (*p* ≤ 0.001) in the presence of caffeine (Figure 5d). Notably, all other analyzed MTXs also showed a highly significant reduction in ROS level in a similar range (theophylline: 54.5% ± 4.8%, *p* ≤ 0.001; pentoxifylline: 48.8% ± 3.2%, *p* ≤ 0.001; theobromine: 39.6% ± 2.7%, *p* ≤ 0.001; propentofylline: 34.4% ± 7.4%, *p* ≤ 0.001) (Supplement Figure S4d). Therefore, all MTXs averaged also revealed a highly significant reduction in ROS level to 45.1% ± 3.6% (*p* ≤ 0.001) (Figure 5d), indicating that MTXs might exert beneficial effects regarding the involvement of oxidative stress in AD pathogenesis.

In addition to oxidative stress, high cholesterol level are discussed to be a risk factor for AD [60,61,62,63,64]. Therefore we analyzed cholesterol level in presence of MTXs using an Amplex red-based cholesterol assay. Caffeine significantly reduced cholesterol level to 82.8% ± 3.7% (*p* ≤ 0.001), whereas the average of all MTXs showed a not significant reduction to 88.0% ± 5.4% (*p* = 0.0892) (Figure 5e). Thereby propentofylline showed the strongest effect on the cholesterol level by a reduction to 70.1% ± 2.8% (*p* ≤ 0.001) (Supplement Figure S4e). On the other hand, theophylline trended to decrease cholesterol level, but statistical analysis did not reach significance (*p* = 0.0520), whereas pentoxifylline and theobromine did not affect the cholesterol level (Supplement Figure S4e).

Additionally, we analyzed whether MTXs affect Aβ42 aggregation using a fluorescent Thioflavin-T-based assay. As positive control we used epigallocatechin gallate, known to inhibit Aβ aggregation [55]. Aβ aggregation was significantly reduced to 46.7% ± 9.6% (*p* ≤ 0.001) in presence of caffeine (Figure 5f). Similarly, all MTXs averaged revealed a significant reduction in Aβ aggregation to 45.6% ± 9.6% (*p* ≤ 0.001) (Figure 5f). In detail, all MTXs individually also showed significantly reduced Aβ aggregation (theophylline: 47.0% ± 9.8%, *p* ≤ 0.001; pentoxifylline: 42.4% ± 10.3%, *p* ≤ 0.001; theobromine: 37.3% ± 7.7%, *p* ≤ 0.001; propentofylline: 54.7% ± 11.8%, *p* = 0.0043) (Supplementary Figure S4f).

## 4. Discussion

AD pathogenesis involves multiple pathological processes, including e.g., Aβ accumulation, tau hyperphosphorylation, oxidative stress, inflammation and alterations in lipid and energy metabolism. Despite intensive research no drug treatment is available to cure AD, only drugs that can help to mask the symptoms. Current research therefore focuses on AD prevention or delay of its progression, including dietary interventions during prodromal and early stages of the disease. Therapeutic strategies that dramatically interfere with a single pathological process involved in AD, e.g., blocking γ-secretase activity, are not reasonable, as γ-secretase dependent cleavage products of APP have important physiological functions and PS can cleave multiple substrates [65,66], probably leading to unwanted adverse effects. Therefore, therapeutic treatments that moderately influence multiple pathological processes are discussed to be more helpful than interventions directed towards a single pathological process involved in AD pathogenesis. MTXs, including caffeine, an adenosine receptor (AR) antagonist, are considered to have health benefits in neurodegenerative diseases, including AD [35,39,67,68,69,70]. Studies investigating caffeine supplementation in AD transgenic mice also revealed that caffeine intake protected against cognitive impairment accompanied by lower hippocampal Aβ level [71]. Similarly, propentofylline prevented learning and memory deficits in β-amyloid infused rats [72], indicating that MTXs may be useful for the treatment of patients with AD. In our present study we therefore examined the mode of action of MTXs on the multiple pathological processes of AD. We selected three naturally occurring MTXs–caffeine, theophylline and theobromine– and two synthetic xanthine derivatives, pentoxifylline and propentofylline. All analyzed MTXs significantly decreased total Aβ level by affecting non-amyloidogenic α-secretase mediated and amyloidogenic β-secretase mediated APP processing. The observed increase in α-secretase processing resulting in elevated sAPPα level in presence of MTXs are caused by an indirect effect due to an increase in the protein stability of ADAM10, the main α-secretase in neurons [24]. MTXs did not affect, or only marginally affected α-secretase activity directly. Simultaneously, amyloidogenic β-secretase processing of APP is decreased as we observed reduced sAPPβ and β-CTF level in MTX-treated cells. Furthermore, MTXs reduced β-secretase activity in living cells, provoked by an indirect effect via decreased *BACE1* gene expression and at least for caffeine by a direct inhibition of β-secretase activity. MTXs therefore induce a shift from β-amyloidogenic to non-amyloidogenic α-secretase processing of APP preventing or reducing the formation of Aβ peptides. These findings are in line with the study by Arendash et al. showing lower hippocampal Aβ level and reduced *BACE1* gene expression in APPswedish transgenic mice supplemented with 1.5 mg caffeine/day per mouse, which is the human equivalent of 500 mg caffeine per day [71] present in five cups of coffee. Reduced amyloid plaque burden was also found when the synthetic MTX propentofylline was fed to APPswedish transgenic mice in a concentration of 40 mg/kg weight per day for one month [73]. In this study propentofylline also attenuated tau hyperphosphorylation, beside Aβ plaques, one of the main pathological hallmarks of AD. Treatment of APPswedish transgenic mice with propentofylline showed an increase in sAPPα level and a decrease in secreted Aβ40 and Aβ42 level [74], which is also in line with our results examining synthetic as well as natural MTXs. Caffeine has also been shown to suppress APP internalization to endosomes [57] where β-secretase processing of APP mainly occurs [75,76]. Notably, suppression of APP internalization simultaneously leads to an APP accumulation at the plasma membrane where APP is processed by the non-amyloidogenic α-secretase [77,78], illustrating a potential therapeutically benefit of MTXs by shifting amyloidogenic to non-amyloidogenic APP processing. Importantly, in our present study we used pure caffeine and not a naturally occurring caffeine source with its other ingredients like ions, caffeic acid, chlorogenic acid and trigonelline, which might act additionally to pure caffeine. Therefore, the effect strength of naturally occurring caffeine e.g., like in coffee consumption, might have a different effect strength than pure caffeine. In this context, e.g., a recent study by Fukuyama et al. is highly interesting. Fukuyama et al. addressed the effect of roasted coffee and its naturally occurring compounds on amyloidogenic β-secretase processing of APP [79]. In line with our findings, they observed by testing roasted coffee (including caffeine, caffeic acid, chlorogenic acid and trigonelline) a reduced BACE1 protein expression accompanied by reduced secreted Aβ level. In their experimental setting, the reduction of Aβ was up to 20%. In our experiments a similar effect strength was observed, showing a reduction of 15.5% of secreted Aβ in presence of pure caffeine. Interestingly, the authors found that also pyrocatechol, produced from chlorogenic acid during roasting, also reduced BACE1 protein expression. These findings suggest that additionally the roasting process and therefore the source of caffeine is crucial for the protective effects of coffee in AD, too. Moreover, it has to be taken into consideration that the pharmacokinetic of caffeine both as a pure substance and by intake from naturally occurring sources, will be different in an in vivo situation compared to cell culture. This might further influence the observed effect strength. However, the observed effects in vivo of caffeine in literature revealed mainly modest effects [69,71,80,81,82], which is in line with the modest effects found in cell culture. We therefore assume that the other MTXs, which are examined here, and which have a similar effect compared to caffeine, have also similar modest effects in vivo. Nevertheless, this has to be proven in further in vivo studies.

Beside the discussed effect of MTXs to reduce the Aβ level by shifting APP processing towards the non-amyloidogenic pathway, we could show that MTXs decrease gene expression of *APP* accompanied by a reduced APP protein level. It is important to mention that the analyzed MTXs reduced APP protein level to approximately 85%, a moderate but significant effect. Taking into consideration, that APP is ubiquitously expressed and highly conserved in any mammalian, it seems reasonable not to completely abolish APP expression and processing, which might be beneficial for treating AD, but have severe side effects by also abolishing a potential physiological function of APP or APP processing.

Interestingly, in our study caffeine and propentofylline also interfered with cholesterol homeostasis, by significantly reducing cholesterol level to 82.8% and 70.1%. Theophylline also showed a trend to decrease cholesterol level. Notably, hypercholesteremia is discussed to be a risk factor for AD and high cholesterol level have been shown to increase Aβ generation in cell culture experiments and mouse models [60,61,62,63,64]. By reducing cholesterol level, MTXs might exert additional beneficial effects regarding Aβ generation and the development of AD. Importantly, it is well established that oxidative stress also plays a crucial role in AD pathogenesis. Aβ induces oxidative stress and in return oxidative stress itself increases Aβ generation [83,84,85]. In the present study, all analyzed MTXs significantly reduced the formation of ROS, probably also contributing to the observed reduced Aβ generation in presence of MTXs, but at least reducing oxidative stress closely linked to AD. Interestingly, it has been shown that oxidative stress stimulates *BACE1* expression [86], which could explain our findings of reduced *BACE1* gene expression in presence of MTXs themselves reducing oxidative stress. Beside the Aβ-reducing effect of MTXs via pleiotropic mechanisms, MTXs also reduce Aβ oligomerization and aggregation, which are essential for the formation of senile plaques. Inhibiting Aβ aggregation is considered as an effective therapeutic strategy to prevent or treat AD. All tested MTXs significantly decreased Aβ aggregation to approximately 40% to 50%. In line, Sharma and colleagues showed that the presence of caffeine in a water solution inhibited the aggregation of Aβ16-22 peptides [87].

Limitations of the study: our present study is a pure in vitro study, utilizing human neuroblastoma SH-SY5Y cells. It aims to clarify the molecular mechanisms how MTXs, especially caffeine, affects processes involved in AD. For the direct effect on β-secretase, results were confirmed ex vivo in mouse brain. Indubitable ex vivo experiments also have their limitations, e.g., no functional blood brain barrier is present or no further metabolization in liver occurs. As discussed above, it is therefore important to emphasize that this study does not include pharmacological or pharmacokinetic aspects, which might alter the effect strength in vivo. However, our results underline that MTXs enhance the non-amyloidogenic pathway and decrease the amyloidogenic pathway resulting in a pleiotropic reduction of Aβ. This reduction has been shown to be modest under the conditions used here and might help to explain why previous in vivo studies also found modest effects in vivo. Additionally an estimation might be made about the other MTXs and their potency to treat or prevent AD, suggesting also only modest effects. However further in vivo studies are needed, to clarify if the molecular mechanisms found here for the other MTXs also lead to comparable effects in respect to AD in a human situation.

## 5. Conclusions

In summary, natural occurring MTXs as well as synthetic xanthine derivatives positively interfere with multiple pathological processes involved in the pathogenesis of AD. The effect strengths on physiological processes like e.g., *APP* gene transcription and APP processing are moderate, but in summary, significantly reduce Aβ generation, and provide the opportunity that APP and its cleavage products can still fulfil their physiological functions. The effect of MTXs on oxidative stress and Aβ aggregation are more pronounced. Therefore, dietary supplementation of MTXs might be a useful therapeutic strategy to prevent or treat AD, as MTXs have pleiotropic positive effects on pathological processes involved in AD pathogenesis. However, due to the observed moderate effect strength, MTXs alone in treating or preventing AD might not be sufficient. Therefore, an integration of MTXs in a dietary supplementation consisting several supplements like DHA, omega three fatty acids, vitamin D, B-vitamins etc. in combination with a healthy life style seems more reasonable and should be taken into consideration, especially due to its cost effectiveness, availability and modest side effects.

Furthermore, our results show that there was no significant difference between the single MTXs on Aβ homeostasis. If the effect on Aβ would be mediated exclusively by adenosine receptor antagonism, it could be assumed that theobromine with lowest affinity to adenosine receptors would have a significant weaker effect compared to the other MTXs, which was not the case but is in line with a pleiotropic action where several mechanisms result in a decreased Aβ level. From this point of view, a further screening for other MTXs which might have even higher affinity to adenosine receptors might have limited additional positive effects in respect to AD. However, although, natural occurring MTXs are rapidly absorbed in the gastrointestinal system, are able to cross the blood-brain-barrier and enter the brain by simple or facilitated diffusion [88,89], further studies are needed to confirm our mechanistically findings in vivo.

## Supplementary Materials

The following are available online at https://www.mdpi.com/2218-273X/9/11/689/s1, Figure S1: Expression of APP^695^ in SH-SY5Y wt and APP^695^ transfected cells, Figure S2: Effects of caffeine, theophylline, pentoxifylline, theobromine and propentofylline on α-secretase, Figure S3: Influence of MTXs on amyloidogenic APP processing, Figure S4: Influence of MTXs on APP level, Aβ catabolism and aggregation, reactive oxygen species and cholesterol, Table S1a: Cytotoxicity by LDH release, Table S1b: Cell proliferation by XTT release.
