# Supplementary material for: Effect of Caffeine and Other Methylxanthines on Aβ-Homeostasis in SH-SY5Y Cells
Source: Biomolecules. 2019 Nov 2;9(11):689. doi: 10.3390/biom9110689 (PMC6920871; doi:10.3390/biom9110689)
Supplement: Supplementary file 1 [file biomolecules-09-00689-s001.pdf]

biomolecules

Supplemental material

Table 1a. Cytotoxicity by LDH release

MTX	mean difference to control [%]
caffeine	-0.54
theophylline	-0.28
pentoxifylline	-0.06
theobromine	1.4
propentofylline	3.78

Table 1b. Cell proliferation by XTT release

MTX	mean difference to control [%]
caffeine	3.8
theophylline	-3.27
pentoxifylline	2.34
theobromine	0.83
propentofylline	-1.91

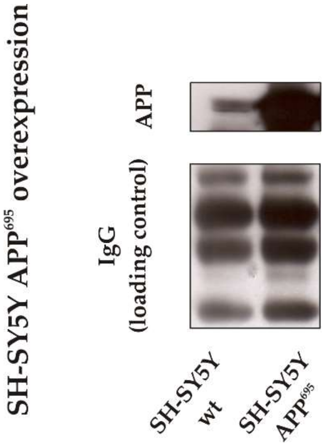

Supplemental Figure 1. Expression of APP^{695} in SH-SY5Y wt and APP^{695} transfected cells

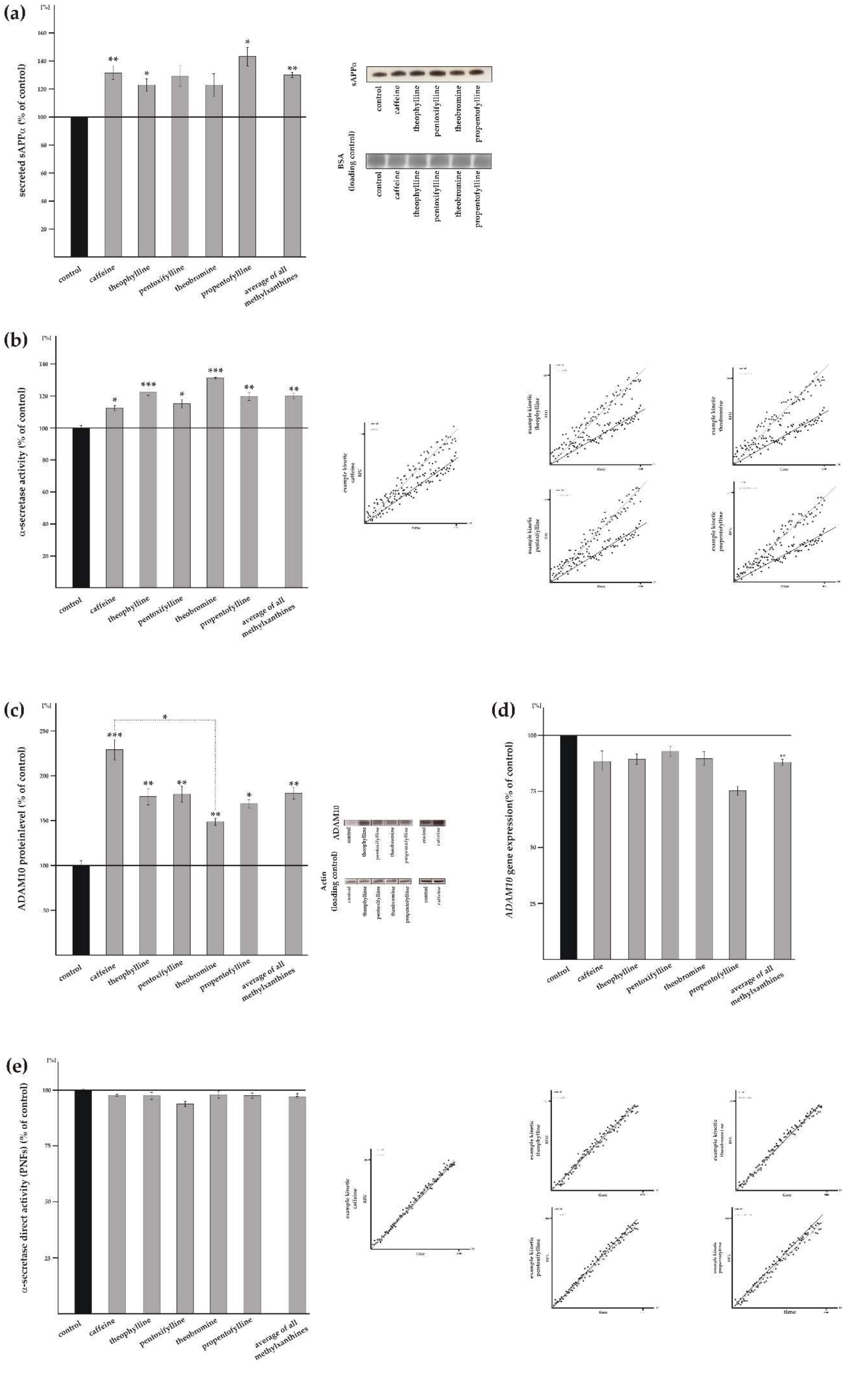
(a)
[%]
secreted sAPPα (% of control)
sAPPα
BSA
(loading control)
control
caffeine
theophylline
pentoxifylline
theobromine
propentofylline
average of all
methylxanthines
(b)
α-secretase activity (% of control)
example kinetic
caffeine
example kinetic
theophylline
example kinetic
theobromine
example kinetic
pentoxifylline
example kinetic
propentofylline
time
(c)
ADAM10 proteinlevel (% of control)
ADAM10
Actin
(loading control)
(d)
ADAM10 gene expression(% of control)
(e)
α-secretase direct activity (PNFs) (% of control)

Supplemental Figure 2. Effects of caffeine, theophylline, pentoxifylline, theobromine and propentofylline on α-secretase. (a) Protein amounts of secreted soluble sAPPα of treated SH-SY5Y cells compared to untreated control cells ($n \geq 9$). Representative WBs including load control are shown on the right. (b) Activity of α-secretase in living SH-SH5Y cells after MTXs treatment ($n \geq 4$). Representative kinetics are shown on the right side. (c) Protein level of ADAM10 ($n \geq 4$). Representative WBs including load control are shown on the right. (d) *ADAM10* gene expression ($n \geq 10$). (e) α-secretase activity in post nuclear fractions of incubated SH-SY5Y cells ($n \geq 4$) and resulting, representative kinetics after MTXs incubation. Error bars represent the standard error of the mean. Asterisks show the statistical significance calculated by unpaired Student's t test (* $p \leq .05$; ** $p \leq .01$; *** $p \leq .001$).

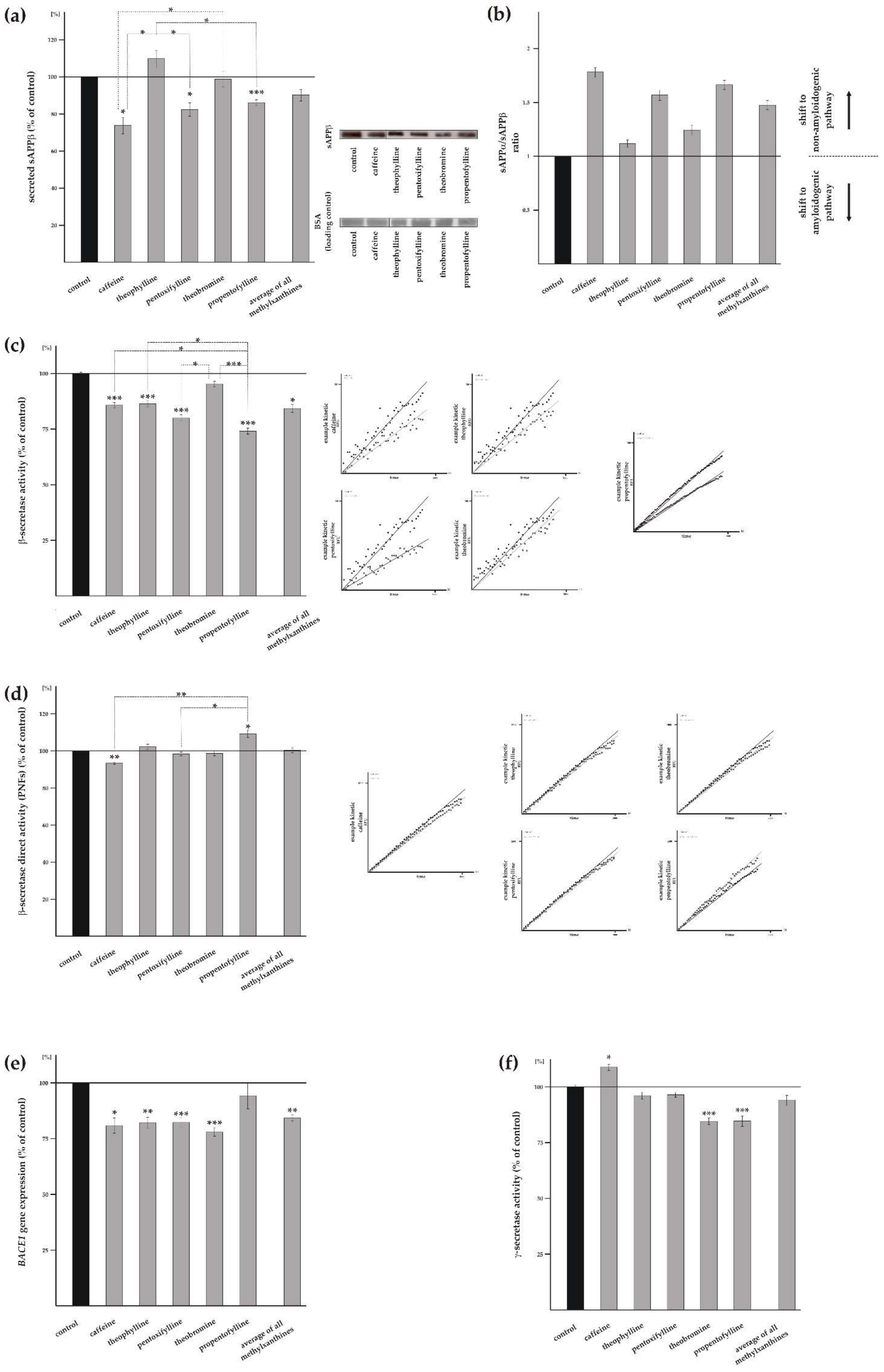
(a)
secreted sAPPβ (% of control)
sAPPβ
BSA (loading control)
control
caffeine
theophylline
pentoxifylline
theobromine
propentofylline
average of all methylxanthines
(b)
sAPPα/sAPPβ ratio
shift to non-amyloidogenic pathway
shift to amyloidogenic pathway
(c)
β-secretase activity (% of control)
example kinetic caffeine
example kinetic theophylline
example kinetic pentoxifylline
example kinetic theobromine
example kinetic propentofylline
time
(d)
β-secretase direct activity (PNFs) (% of control)
(e)
BACE1 gene expression (% of control)
(f)
γ-secretase activity (% of control)

Supplemental Figure 3. Influence of MTXs on amyloidogenic APP processing. (a) Protein level of sAPPβ of treated SH-SY5Y APP[695] cells (n ≥ 13). Representative WBs including load control are shown on the right. (b) sAPPα/sAPPβ ratios. (c) β-secretase activity (n ≥ 19). Representative kinetics for each analyzed MTX are shown on the right. (d) Analysis of the activity of β-secretase in post nuclear fractions from SH-SY5Y cells (n ≥ 6). Example kinetics are illustrated on the right side. (e) Level of *BACE1* mRNA (n ≥ 10). (f) Activity of the γ-secretase (n ≥ 25). Error bars represent the standard error of the mean. Asterisks show the statistical significance calculated by unpaired Student's t test (* p ≤ .05; ** p ≤ .01; *** p ≤ .001).

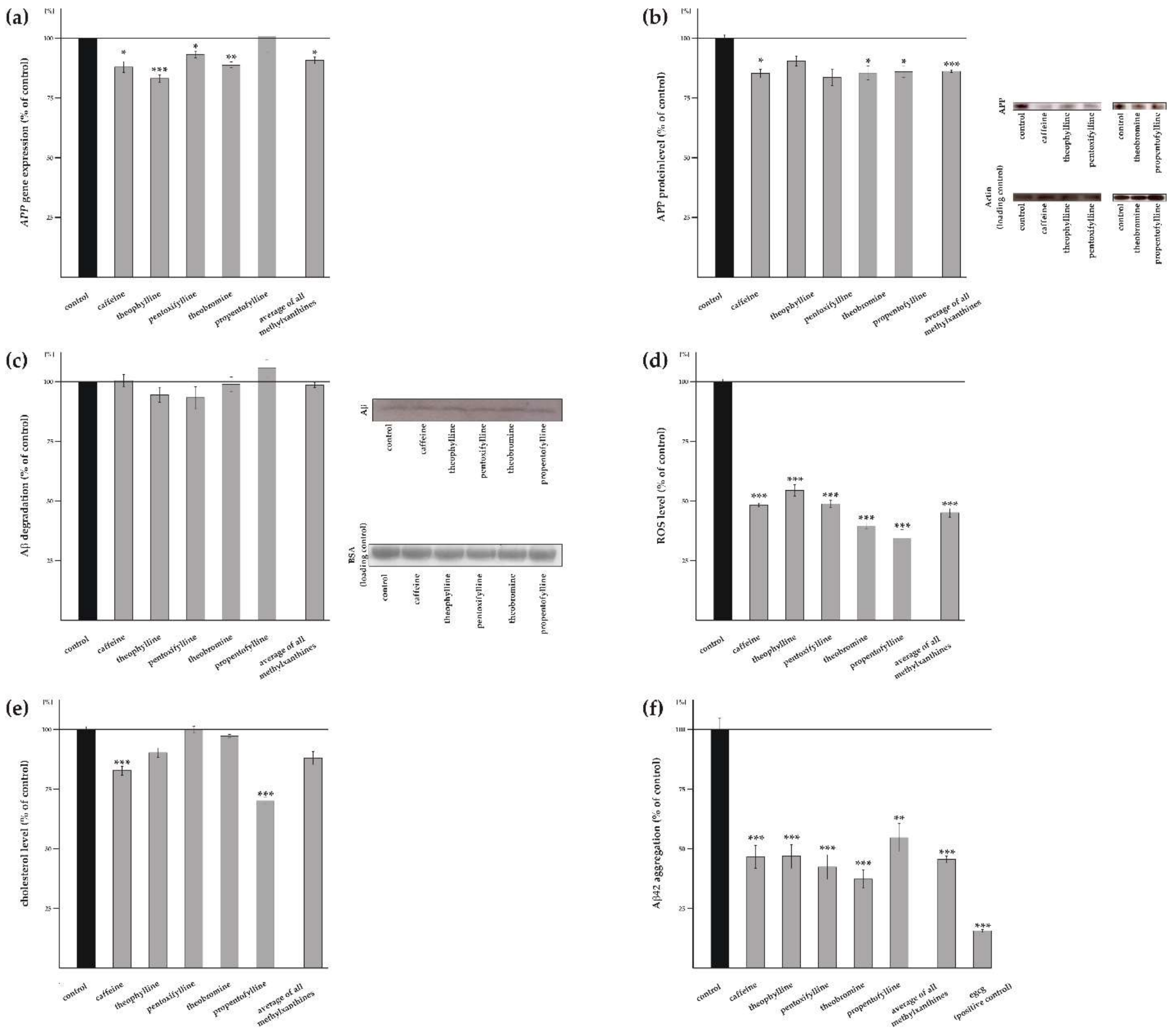

Supplemental Figure 4. Influence of MTXs on APP level, Aβ catabolism and aggregation, reactive oxygen species and cholesterol. (a) Gene expression of *APP* (n ≥ 10). (b) APP protein level in lysates of treated SH-SY5Y cells (n ≥ 6). Representative WBs including load control are shown on the right. (c) Influence of MTXs on ROS level in human neuroblastoma cells (n ≥ 8). (d) Aβ degradation in the presence of MTXs (n ≥ 11). Representative WBs including load control are shown on the right. (e) Effect of MTXs on cholesterol level (n ≥ 4). (f) Influence of MTXs on Aβ42 aggregation (n ≥ 20). Epigallocatechin gallate (EGCG) served as positive control. Error bars represent the standard error of the mean. Asterisks show the statistical significance calculated by unpaired Student's t test (* p ≤ .05; ** p ≤ .01; *** p ≤ .001).
